# Long-Term Pharmacokinetic Follow-Up of Abiraterone Acetate in Patients with Metastatic Castration-Resistant Prostate Cancer

**DOI:** 10.3390/ijms25116058

**Published:** 2024-05-31

**Authors:** Emmanuel Chamorey, Marc Pujalte-Martin, Jean-Marc Ferrero, Hakim Mahammedi, Gwenaelle Gravis, Guilhem Roubaud, Philippe Beuzeboc, Remy Largillier, Delphine Borchiellini, Claude Linassier, Hélène Bouges, Marie-Christine Etienne-Grimaldi, Renaud Schiappa, Jocelyn Gal, Gérard Milano

**Affiliations:** 1 Epidemiology and Biostatistics Department, Centre Antoine Lacassagne, University Côte d’Azur, 06000 Nice, Francejocelyn.gal@nice.unicancer.fr (J.G.); 2 Medical Oncology Department, Centre Antoine Lacassagne, University Côte d’Azur, 06000 Nice, France; 3 Medical Oncology Department, Centre Jean Perrin, 63000 Clermond Ferrand, France; 4 Medical Oncology Department, Institut Paoli Calmette, 13009 Marseille, France; 5 Department of Medical Oncology, Institut Bergonié, 33076 Bordeaux, France; 6 Medical Oncology Department, Institut Curie, 75005 Paris, France; 7 Medical Oncology Department, Centre Azuréen de Cancérologie, 06250 Mougins, France; 8 Clinical Research Department, Centre Antoine Lacassagne, University Côte d’Azur, 06000 Nice, France; 9 Medical Oncology Department, Centre Hospitalier Régional Universitaire, 37000 Tours, France; 10 Oncopharmacology Unit, Centre Antoine Lacassagne, University Côte d’Azur, 06189 Nice, France

**Keywords:** prostate cancer, abiraterone acetate, pharmacodynamics, pharmacogenetics

## Abstract

This ABIGENE pharmacokinetic (PK) study sought mainly to characterize the unchanged drug PK during long-term abiraterone acetate (AA) administration in advanced prostate cancer patients (81 patients). It was observed that individual AA concentrations remained constant over treatment time, with no noticeable changes during repeated long-term drug administration for up to 120 days. There was no correlation between AA concentrations and survival outcomes. However, a significant association between higher AA concentrations and better clinical benefit was observed (*p* = 0.041). The safety data did not correlate with the AA PK data. A significant positive correlation (r = 0.40, *p* < 0.001) was observed between mean AA concentration and patient age: the older the patient, the higher the AA concentration. Patient age was found to impact steady-state AA concentration: the older the patient, the higher the mean AA concentration. Altogether, these data may help to guide future research and clinical trials in order to maximize the benefits of AA metastatic castration-resistant prostate cancer patients.

## 1. Introduction 

Metastatic castration prostate cancer (mCRPC) is responsible for the majority of prostate cancer deaths. Taxane-based chemotherapy is the cornerstone of the treatment options for mCRPC [1]. In 2011, abiraterone acetate (AA) plus prednisone became the first new anti-androgen treatment to be introduced in this setting, and it has significantly improved the field of prostate cancer treatment. Following advances in chemotherapy, AA plus prednisone improved overall survival compared to placebos plus prednisone, with a hazard ratio of 0.65 [95% CI [0.54–0.77]; *p* < 0.001] [2]. In addition to improving survival, AA also delays skeletal-related events and improves quality of life compared to placebos [3].

AA inhibits cytochrome *CYP17A1*, which provides 17alpha-hydroxylase activity. This form of cytochrome is present in testicular, adrenal cortex and prostate tumors. The inhibition of *CYP17A1* stops the biosynthesis of androgens, testosterone, dihydrotestosterone and cortisol. Abiraterone, given orally as an acetate prodrug, has effectively decreased testosterone in non-castrated males by approximately 50% [4]. A successful therapy of AA may pose some questions related to the prodrug nature of AA and its possible inter-individual variability in bioavailability and blood pharmacokinetics. A relatively limited current understanding of the pharmacokinetics (PKs) of AA suggests that inter-individual variability for the PKs of AA exists and may influence PSA response and progression-free survival [5]. However, this PK knowledge about AA remains limited, particularly with regard to long-term follow-up. Such information is of potential importance for a drug such as AA that is administered orally and on a long-term basis.

The main objective of this prospective multicenter ABIGENE study was to investigate the pharmacological–biological relationships associated with AA treatment using three main applications: pharmacogenetics, PKs and hormonal monitoring. The three separate parts were planned to be published independently. This pharmacogenetic study indicated that the gene polymorphism of *CYP17A1* (rs743572) may be associated with the efficacy of AA treatment and may identify patients at risk for toxicity [6]. More precisely, from a multivariate analysis, the rs743572 and performance status were independently associated with significant toxicity. The findings suggested that host genome characteristics may help to predict AA treatment pharmacodynamics. In complement to the ABIGENE pharmacogenetic study, the present ABIGENE PK study is mainly dedicated to PK characterization during long-term AA administration in advanced prostate cancer patients. In addition, potential PK/pharmacodynamic (PD) relationships were also examined. 

## 2. Results

### 2.1. Study Patients

Of the 148 patients in the ABIGENE study, 81 patients provided plasma samples and were included in the PK analysis. The main patient characteristics are depicted in Table 1. The mean age was 72 years (7.92). The performance status was 0 or 1 in 97.5% of patients. The primary site of metastasis was bone (81.5%). Most patients (49.5%) had Gleason scores of 7. The median PSA was at 28.5 ng/mL (10 to 52).

### 2.2. Treatment Efficacy 

The radiographic responses were complete in 2 patients (2.5%) and partial in 10 patients (12.7%). Sixty-four patients (81.0%) had stable disease and three patients (3.8%) progressive disease. Seventy-six patients (96.2%) were in clinical benefit. In terms of biological response, 51 patients had a complete biological response (63.75%). The PSA-50 response rate was achieved in 61 patients (76.25%). The median follow-up was 59.1 months (95% CI [53.4–61.4]). The 3-year and 5-year OSs were 52% [42.0–64.0] and 22% [14.0–35.0], respectively; the 3-year rPFS was 14% [8.0–24.0] and the bPFS was 11% [6.0–21.0]. The median OS and rPFS were 37.5 months (95% CI [31.9–44.6]) and 16 months (95% CI [13.5–22.7]), respectively. 

### 2.3. Safety

Fifty patients (61.7%) experienced at least one treatment-related toxicity event. Among these 50 patients, 188 adverse events were identified, of which 169 were Grade 1/2 (90%) and 19 were Grade 3/4 (10%). The most common Grade 3/4 treatment-related toxicities were hypertension (n = 2), asthenia (n = 2), increased alkaline phosphatase (ALP) (n = 2) and abdominal pain (AAT) (n = 2). 

### 2.4. Descriptive Analysis of PK Profiles

**Figure 1:**
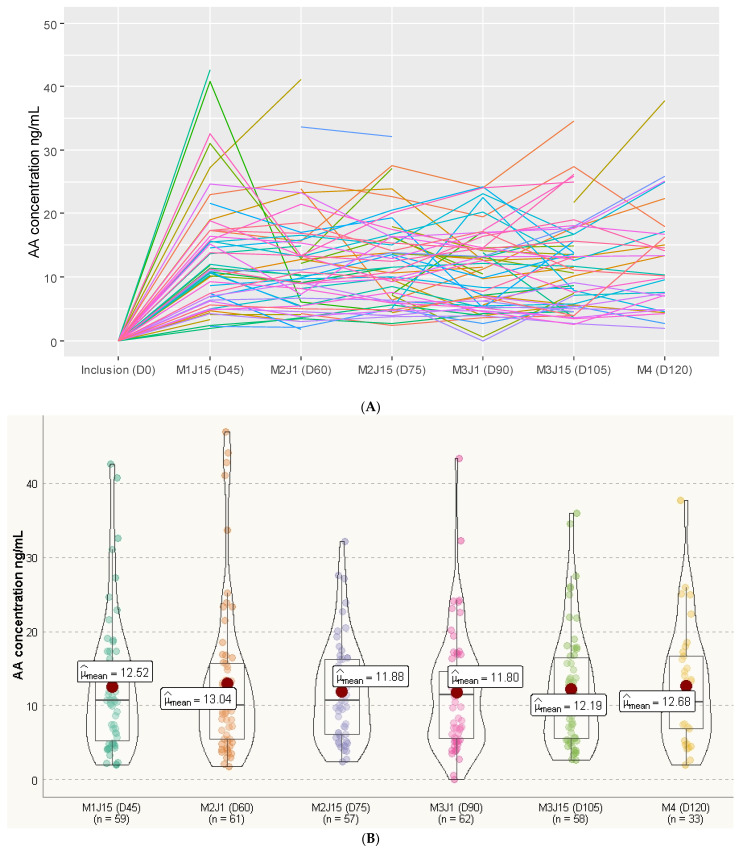
Individual AA plasma concentration evolution during treatment time. (**A**) Evolution of individual residual of AA concentrations (C_min_) during the treatment course. (**B**) Evolution of the means of individual C_min_ AA concentrations during the treatment course.

**Figure 2:**
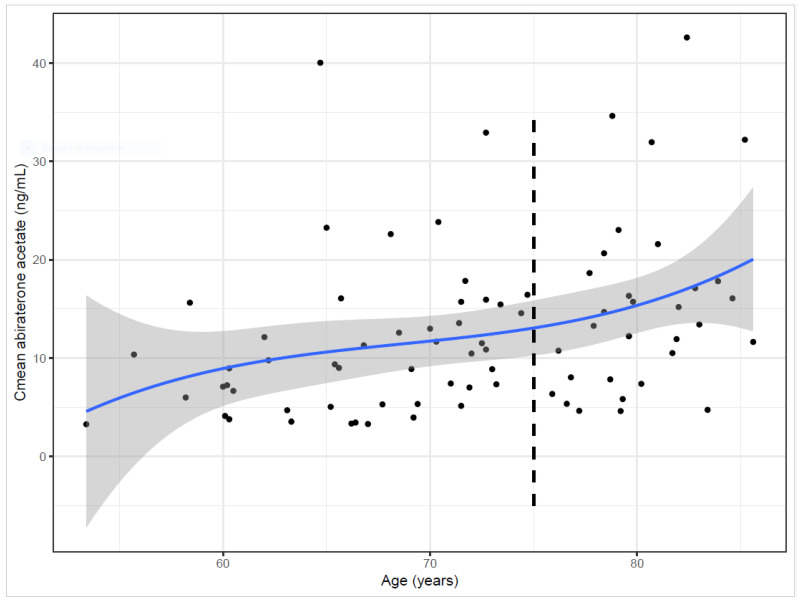
Relationship between patient age and individual AA C_mean_ values. A second-degree polynomial regression (blue line with 95% CI; $y=12.9-0.0391x+0.000532x^{2}$; R^2^ = 0.16; *p* < 0.001) best identified the correlation between patient age and AA pharmacokinetics (C_mean_ values). A threshold was identified at 75 years old (position of the vertical dashed line), in agreement with the EDE method (see Section 4), for a greater impact of age on AA pharmacokinetics.

Of the 81 patients in this study, 22 patients (27.2%) had complete profiles of PK data (i.e., six C_min_ measurements), 19 patients (23.5%) had five C_min_ measurements, 13 patients (16.0%) had four C_min_ measurements, 10 patients (12.3%) had three C_min_ measurements, 12 patients (14.8%) had two C_min_ measurements and 5 patients (6.2%) had one C_min_ measurement. The average (±SD) C_min_ values at each sampling time were 12.52 ± 9.01 ng/mL at D45, 13.04 ± 10.45 ng/mL at D60, 11.88 ± 6.72 ng/mL at D75, 11.80 ± 7.84 ng/mL at D90, 12.19 ± 7.85 ng/mL at D105 and 12.68 ± 8.06 ng/mL at D120 (Figure 1). When the mean values of C_min_ at each time point were considered, no statistically significant effect of time was observed on the evolution of the mean concentrations during follow-up (*p* = 0.97). Importantly, there was a global stability over time of the AA C_mean_ (Figure 1B). This observation concurs well with the overall maintenance of the individual AA C_min_ during the follow-up (Figure 1A). No significant correlation was observed between the individual AA C_mean_ of each patient and liver function markers (gamma glutamyl transferase, ASAT, ALAT, alkaline phosphatase and bilirubin). Although the correlations between the AA C_mean_ values and the tested parameters were non-significant (Table 2), gamma glutamyl transferase (GGT) exhibited a slight tendency toward a link with AA C_mean_ (r = 0.15, *p* = 0.16). Of note, a significant positive correlation (r = 0.40, *p* < 0.001) was observed between mean AA concentration and patient age: the older the patient, the higher the AA concentration. As shown in Figure 2, statistical analyses identified a second-degree polynomial regression model depicting this relationship between age and AA PKs and a threshold above 75 years old for a greater impact of aging on individual AA C_mean_ concentrations.

### 2.5. Pharmacokinetic–Pharmacodynamic Relationships

As shown in Table 3, considering the 50 patients with at least one toxic event, there was no significant correlation (*p* = 0.25) between patient C_mean_ and grade of toxicity (Grade 1/2 vs. Grade 3/4). 

However, interestingly, there was a significant relationship between the individual AA concentration profiles and radiographic clinical benefit, as shown when considering the AA C_mean_ values between patients with progressive disease (median: 3.95 ng/mL [IQR: 3.65–6.47]) and others (CR/PR/SD median: 11.78 ng/mL [IQR: 7.29–16.05]; *p* = 0.04). There were no significant relationships between individual C_mean_ and PSA-50 response rate. Survival analyses were performed using a threshold set as the AA C_mean_ (12.75 ng/mL). There was no significant difference in OS, rPFS or bPFS according to this threshold. 

## 3. Discussion

To our knowledge, the ABIGENE project is one of the first to investigate the long-term PK profile of AA in patients treated for mCRPC in real-word clinical practice. This study did not show an association between AA concentrations and survival outcomes. However, a significant association was observed with higher AA concentrations in terms of being associated with better clinical benefit. Similar associations have been previously reported for AA concentration, PSA response and PFS [5]. Thus, in a population pharmacokinetics analysis of AA, Stuyckens and colleagues [7] mentioned that AA exposure significantly impacted PSA elimination. A review by Benoist and coworkers [8] indicated that an association had been shown between AA concentration, decreased PSA and increased survival. This information was confirmed by the data reported by Carton and coworkers [5], the authors proposing that AA concentration monitoring could be a valuable approach to improve clinical outcomes in castration-resistant prostate cancer. Then, after, Blanchet and coworkers reported on an AA metabolite survey and mentioned that a high metabolic ratio could help to identify patients with increased activity of the main AA metabolizing enzyme and who were at risk for a poorer survival rate [9]. The existence of an exposure threshold of AA concentration linked to survival was also underlined by the data reported by Boerrigter and coworkers [10]. Therefore, it is reasonable to assume that AA PKs may reflect treatment outcome, and this relationship should be strengthened by studies based on a large number of patients. The safety data from this ABIGENE PK study were not correlated with AA PKs. A similar conclusion was reached in a monocentric prospective observational study when the incidence of adverse events was compared to the minimum steady-state concentrations of AA [5].

Notably, this study found in its main objective that individual AA concentrations remained statistically constant over time, with no noticeable changes during the long-term administration of the drug for up to 120 days. These relatively constant concentrations over time may reflect good compliance and bioavailability of the treatment. Stable AA concentrations during a long-term treatment course (median follow-up: nearly 60 months) have ruled out the hypothesis of a metabolic resistance phenomenon during long-term treatment with AA. This mechanism of resistance has been suggested to be due to the induction of drug-metabolizing enzymes over time [11]. Narenda and coworkers [12] insisted on the role of single-nucleotide polymorphisms in drug-metalizing enzymes by modifying the profiles of resistant anti-cancer drugs.

Interestingly, patient age was found to be associated with steady-state AA concentration. The older the patient, the higher the mean AA concentration. The present data contrast with those from a retrospective study by Crombag et al. [13], in which no such association between age and AA concentration was observed. As AA is subject to marked hepatic metabolism [9], the known decline in liver function with age may help explain why individual AA concentrations increase with age due to a loss of individual metabolic capacity, which is consistent with the present data. A possible explanation for the discrepancy between the present data and those reported by Crombag et al. [13] is the fact that the present study dichotomized the effect of age. The significant positive association between age and AA concentration (Figure 2) is consistent with the notion of a positive age-related decline in hepatic metabolism [14]. Less metabolism translates into more unaltered drug circulation. It was beyond the scope of the present study to perform an analysis of AA metabolism, but this point should be explored in light of the present results. The present study identified an age cut-off of 75 years, which was also pointed out by Mulders et al. [15] and which is consistent with the knowledge of the impact of advanced age on oral drug absorption [16], where a reduction in liver mass and blood flow may intervene [17]. It is interesting to note in this respect that prostate cancer patients over 75 years of age derive significant benefit from AA treatment [18]. Among the tested parameters of the hepatic function, only GGT levels at baseline exhibited a slight tendency toward a correlation with the AA C_mean_. GGT is a cell surface enzyme that hydrolyzes the gamma-glutamyl bonds of extracellular reduced and oxidized glutathione, initiating their cleavage into glutamate, cysteine and glycine. GGT is a well-established serum marker of hepatobiliary disease. Several studies have shown an association between GGT levels and cardiovascular risk [19] as blood cholesterol levels rise [20,21]. AA and cholesterol share a similar chemical structure. AA has four carbon cycles of cholesterol with an aromatic pyridine cycle and an acetate linkage. We can hypothesize that the liver, which is involved in the absorption of cholesterol, may increase the bioavailability of AA when GGT concentrations are elevated in accordance with liver dysfunction, showing a lower absorption capacity. Another explanation for the association of higher AA concentrations with high GGT levels is the possibility that a loss of hepatic metabolic capacity may correspond to high GGT levels.

## 4. Materials and Methods

### 4.1. Study Design and Patients

ABIGENE was an open-label, prospective, multicenter trial that included a cohort of 148 patients and was conducted between February 2014 and June 2017 across 19 sites. Inclusion criteria were an age of 18 years or older with a histologically confirmed prostate adenocarcinoma with evidence of metastatic disease on androgen deprivation therapy (ADT), with serum testosterone levels ≤ 50 ng/mL. Patients were required to have an Eastern Cooperative Oncology Group performance status ≤ 2 and adequate organ bone marrow function. Major exclusion criteria included prior cytotoxic treatment, prior AA treatment and known hypersensitivity or allergy to AA or any of the excipients. According to routine practice, AA (1000 mg) and prednisone (10 mg) were administered daily until unacceptable toxicity or disease progression. Written informed consent was obtained from each patient. The French Institutional Ethics Committee approved this study in 2012 (NCT01858441).

### 4.2. Pharmacokinetic Measurements

Pharmacokinetic analyses were performed on 81 patients. Blood samples for PK analysis were collected at enrollment (D0), at day 45 (M1J15) and every 15 days thereafter: the 60th day (M2J1), 75th day (M2J15), 90th day (M3J1), 105th day (M3J15) and 120th day (M4) after treatment initiation and a final one at the last visit. The blood samples were collected in the morning before daily drug intake (C_min_). Given the longitudinal design of this study, for each patient, the AA exposure was defined as the mean of all available AA residual concentrations (C_mean_). Using the interquartile range (IQR) method [22], eleven C_min_ concentrations were identified as outliers and, as such, thus removed from the analysis (ranging from 52.15 ng/mL to 138.40 ng/mL). 

The AA was quantified by high-performance high-pressure liquid chromatography coupled with mass spectrometry derived from a previously described analytical method [23]. The limit of quantification was 1 ng/mL.

### 4.3. Pharmacodynamic Measurements

The pharmacodynamics-related parameters for AA were biological progression-free survival (bPFS), radiographic progression-free survival (rPFS), overall survival (OS) and toxicity. Disease progression was defined as radiographic progression in soft tissue or bone. Radiographic disease progression assessment was defined by at least one of the following conditions: (1) a progression on a CT scan, according to the Response Evaluation Criteria in Solid Tumors version 1.1 (RECIST 1.1), or (2) a progression on a bone scan with the appearance of ≥2 new lesions during ADT, according to Prostate Cancer Clinical trials Working Group 2 (PCWG2) [24]. The PSA was measured at baseline and every 4 weeks. The clinical benefit was defined as the proportion of patients exhibiting a complete response (CR) or partial response (PR), including stable disease (SD), and was assessed by an enhanced CT scan according to the RECIST 1.1 criteria or a bone scan according to criteria adapted from the PCWG2 criterion [24]. Imaging was performed at baseline and every 12 weeks thereafter. A biological response based on the PSA was defined as a decrease of at least 50% from the baseline value (PSA-50), while PSA progression was defined as an increase of 50% from the nadir. Biological progression-free survival (bPFS) was defined as the time from treatment initiation to PSA progression. Radiographic progression-free survival (rPFS) was defined as the time from treatment initiation to radiographic progression. Overall survival (OS) was defined as the time from treatment initiation to death from any cause. Patients were censored at the dates they were last known to be alive if they had no events (death or progression) or were lost to follow-up. Treatment-related toxicity was assessed using the Common Terminology Criteria for Adverse Events (CTCAE 4.0). A serious adverse event was defined as the occurrence of any Grade 3/4 toxicity.

### 4.4. Statistical Analysis

Descriptive statistics were used to summarize the demographic and clinical characteristics. Frequency distributions and percentages were used to summarize the categorical variables, and medians with ranges were used to describe the continuous variables. When data are not available, they are considered missing data. Missing data are presented as numbers and percentages. Comparisons of continuous variables between groups were assessed by a 2-sample *t*-test or by an analysis of variance for more than 2 groups, if normality was assumed. Otherwise, the appropriate non-parametric equivalents, the Wilcoxon rank-sum test, Mann–Whitney and Kruskal–Wallis test, were used. bPFS, rPFS and OS were estimated using the Kaplan–Meier method and are presented on an indicative basis only. Censored data were presented with survival at various time points, median survival and a 95% confidence interval. Median follow-up was calculated using the inverse Kaplan–Meier method. Survival curves were compared using the log-rank test. The correlation between C_mean_ and each analyzed continuous covariate (age, as well as ASAT, ALAT, GGT, alkaline phosphatase and bilirubin measured at baseline) was analyzed using the non-parametric Spearman test. The association between AA C_mean_ and age was analyzed according to an adapted statistical computing method giving the nature of the regression and the coefficient of determination (R²). In addition, independently, the identification of a possible threshold for the impact of age on AA PKs was tested using Extremum Distance Estimator (EDE) methods [25]. Statistical significance was declared at α ≤ 0.05, and no adjustments were made for multiple testing. All statistical analyses were performed with the statistical computing software R (https://www.r-project.org), version 4.1.1, on Windows^®^.

## 5. Conclusions

The ABIGENE PK study reported here may provide useful information regarding the long-term PKs of AA in the treatment of mCRPC. The study results suggest that AA concentrations remain stable with long-term administration and may be associated with improved biological responses. The observed associations between AA PKs, patient age and GGT levels warrant further investigation to better understand the factors influencing AA exposure. Attempts are now being made to synergize the effects of AA by activating other pathways. Recently, the combination of AA with olaparib (a poly (ADP-ribose) polymerase inhibitor) in mCRPC patients was approved by the FDA [26], with an improvement in survival especially in BRCA-mutated patients. Taken together, accumulated PK knowledge of AA may help to optimize AA dosing and to design future treatment strategies in order to improve therapeutic outcomes for patients with mCRPC.

## Figures and Tables

**Table 1:** Main patient characteristics at baseline and treatment response.

Characteristics	N° of Patients (n = 81)	Missing Data
Age (years) _mean (SD)_	72 (7.92)	0 (0%)
<75 years	49 (60.5%)	
≥75 years	32 (39.5%)	
Body weight (kg) _median [Q1–Q3]_	80 [57.0–115.1]	5 (6%)
Body mass index _median [Q1–Q3]_	26.7 [21.03–37.86]	5 (6%)
Baseline PSA (ng/mL) _median [Q1–Q3]_	28.5 [10.0–52.0]	0 (0%)
Metastatic disease at diagnosis		12 (14.8%)
Not metastatic	50 (72.5%)	
Metastatic	19 (27.5%)	
Site of metastases, n (%)		0 (0%)
Bone	66 (81.5%)	
Lymph node	43 (53.0%)	
Lung	10 (12.3%)	
Other	7 (8.6%)	
Gleason score		4 (5%)
≤6	8 (10.5%)	
7	38 (49.5%)	
≥8	31 (40.0%)	
Baseline gamma-glutamyl transferase (UI/l) _median [Q1–Q3]_	28.5 [20.75–46.25]	1 (1.25%)
Baseline ASAT (UI/l) _median [Q1–Q3]_	21 [17.0–25.0]	0 (0%)
Baseline ALAT (UI/l) _median [Q1–Q3]_	18 [14.0–23.0]	0 (0%)
Baseline bilirubin (µmol/l) _median [Q1–Q3]_	8.6 [6.0–11.0]	0 (0%)
Baseline alkaline phosphatase (UI/l) _median [Q1–Q3]_	89 [66.0–129.0]	4 (4.9%)
Performance status		4 (4.8%)
0	39 (50.5%)	
1	36 (47.0%)	
2	2 (2.5%)	
Treatment-related toxicity		0 (0%)
Grade 1/2	169 (90%)	
Grade 3/4	19 (10%)	
Radiographic response		2 (2.5%)
Complete response	2 (2.5%)	
Partial response	10 (12.7%)	
Stable disease	64 (81.0%)	
Progressive disease	3 (3.8%)	
Biological response		1 (1.25%)
Biological complete response	51 (63.75%)	
Biological response	10 (12.5%)	
No biological response	12 (15.0%)	
Biological progression	7 (8.75%)	

**Table 2:** Correlations between individual AA C_mean_ and hepatic function markers.

GGT	ASAT	ALAT	Alkaline Phosphatase	Bilirubin
r = 0.15	r = 0.1	r = −0.02	r = −0.06	r = 0.07
*p* = 0.16	*p* = 0.4	*p* = 0.8	*p* = 0.6	*p* = 0.5

Table keys: GGT = gamma glutamyl transferase; ASAT = aspartate aminotransferase; ALAT = alanine aminotransferase. This table depicts the respective Spearman coefficients of correlation between each considered variable of the hepatic function and the individual AA C_mean_.

**Table 3:** Individual AA C_mean_ as a function of treatment pharmacodynamics.

Treatment-Related Toxicity	Grade 1/2 (N = 169)	Grade 3/4 (N = 19)	*p*-Value *
	12.47 ng/mL [7.22–17.1] **	9.4 ng/mL [5.34–17.06] **	0.257
**Clinical benefit**	**CR/PR/SD (N = 76)**	**PD (N = 3)**	*p*-value *
	11.78 ng/mL [7.29–16.05] **	3.95 ng/mL [3.65–6.47] **	0.041

Table keys: CR = complete response, PR = partial response, SD = stable disease, PD = progressive disease; * Wilcoxon signed-rank test for group comparisons (*p*-value); ** median [Q1–Q3].

## Data Availability

Individual participant data are available on reasonable request where research proposals have ethical approval. Participant data will be anonymized and only available for participants consenting to data sharing.
